# Microbial contamination data of keypad and touch screen of cell phones among hospital and non-hospital staffs – A case study: Iran

**DOI:** 10.1016/j.dib.2018.07.041

**Published:** 2018-07-27

**Authors:** Amin Dorost, Yayha Safari, Maliheh Akhlaghi, Marzieh Soleimani, Nasrin Yoosefpour

**Affiliations:** a Students Research Committee, Torbat Heydariyeh University of Medical Sciences, Torbat Heydariyeh, Iran; b Research Center for Environmental Determinants of Health, Kermanshah University of Medical Sciences, Kermanshah, Iran; c Student of Environmental Health Engineering, Health Faculty, Students Research Committee, Gonabad University of Medical Sciences, Gonabad, Iran; d Students Research Committee, School of Public Health, Kermanshah University of Medical Sciences, Kermanshah, Iran

**Keywords:** Bacteria contamination, Keypad phone, Touch screen phone, Cell phone, Hospital, Gonabad, Iran

## Abstract

Microorganisms live almost everywhere, they are even present on inanimate objects such as Mobile phones, as a result contaminates our body. The main purpose of this study was tantamount to compare microbial contamination of keypad and touch screen mobile cell phones between hospital and non-hospital staffs. Samples were collected from 456 cell phones of hospital and non-hospital. Microbial swab samples were taken from 1 cm^2^ of surface from each cell phone, and incubated on Brain Heart Infusion agar media at 37.5 °C for 24 h. Isolated microorganisms were grown aerobically on 55% defibrinated Sheep Blood and eosin methylene blue agar media at 37.5 °C for 48 h. In present study the antibiotic microorganism-resistant could not be observed. Overall, 456 cell phones were collected: 240 (52.63%) from hospital staff (nurses), 216 (47.36%) from non-hospital staff (health care worker outside the hospital). The result indicates that the bacterial contamination of phones used by all of different investigated groups was lower in touch screen devices than keypad devices and the contamination was found more in hospital staff cellphone than non-hospital staff׳s cell device. Woman׳s cell also has a few colonies rather than man׳s cell phones. The dominant microorganisms in the hospital staff were, *Enterobacteriaceae*, Bacillus species, especially Gram-positive bacteria sporulated and staphylococcal negative coagulase, respectively. Cell phones could be a serious threat to the spread of cross-infection in hospitals, therefore development of hand hygiene and cell phone cleaning guidelines is needed regarding public cell phone use.

**Specifications Table**

**Table:** 

Subject area	Environmental health science
More specific subject area	Environmental microbiology
Type of data	Tables
How data was acquired	In this study, the 456 samples of cell phones were collected from hospital and non-hospital (one educational hospital). Microbial samples were taken from 1 cm^2^ surfaces of the cell phones using a sterile swab, and incubated on Brain Heart Infusion agar media at 37.5 °C for 24 h.
Data format	Raw, analyzed
Experimental factors	All isolated microorganisms were grown aerobically on 55% defibrinated Sheep Blood and eosin methylene blue agar media at 37.5 °C for 48 h. Isolated microorganisms were identified using Gram׳s staining, colony morphology and appropriate biochemical procedures
Experimental features	All sampling and microbial analysis were performed according to the standard method of microbial tests.
Data source location	Gonabad city, Iran
Data accessibility	Data are included in this article

**Value of the data**

• The cause of the disease, as well as a range of environmental microorganisms [1], [2], [3], [4], [5], [6], [7], [8]. The data of this study examined the above-mentioned issue.
• So far fewer studies have been made in Iran. Accordingly, the data of the present study can be used for current plication, moreover it can be the basis for future studies.
• In Iran, the issue of transmission of bacterial contamination via mobile phones is investigated in a few researches. The data from this study could give more attention to this problem.
• The data of this study showed that mobile phones are a serious threat to the spread of cross-infection in hospitals. So, strongly recommended that healthcare worker and patients׳ companions have limited use of mobile phones in clinical sectors, especially in special and high-risk sectors.

## 1 Data

In Table 1, the number of bacterial colonies has been shown in the sample collected from male and female staff in the hospital and the control group. The count of colonies is shown in Figure 1, high count of 72 colonies belongs to the samples of untouched phone devices used by man hospital staff and low count of 5 colonies belong to collected samples of the touch phones used by the control group in the female. Also, the results of linear regression analysis showed that the logarithm of colony numbers is significantly affected by the type of phone variable (*R* = 0.86).

**Table 1:** Bacterial colonies in hospital staff and control samples (CFU after 48 h).

**Sex**	**Number**	**Groups**	**Type**	**Number**	**Percent**	**Mean**	**SD**
Male	214	Hospital	Touch	50	10.96	46.5	32.35
			Key	67	14.69	71.64	20.4
		Evidence	Touch	45	9.86	8.56	9.43
			Key	52	11.4	16.49	4.52
Female	242	Hospital	Touch	12.06	14.91	26.76	26.3
			Key	13.37	12.06	41.85	15.39
		Evidence	Touch	12.71	13.37	4.82	6.2
			Key	12.06	12.71	12.7	8.42

Table 2 shows the average number of bacterial colonies in hospital staff and the control sample in terms of bacterial counted colonies in 48 h. Table 2 shows that the average number of bacteria counted colonies after cultivation in the incubator for 48 h was 446.67 colonies in the hospital staff and 10.64 colonies in the control sample. Results of Kruskal Wallis analyzed showed that there was a significant difference between the number of colonies counted in all models of phones of hospital staff and control samples, and the number of colonies was more in hospital samples (*P* = 0.020).

**Table 2:** Average of bacterial colonies in hospital staff phone samples (CFU 48 h).

**Descriptive parameters**	**Hospital staff**	**Non-hospital staff**
Samples	240	216
Mean	46.67	10.64
Median	12	5
SD	24.10	10.25
Min	0	0
Max	405	46

The average number of bacterial colonies in both touch-screen and untouched phones is provided in Table 3. The average number of bacterial colonies in touch-screen and mechanical phones was 21.66 and 35.67 CFU, respectively. The results of Kruskal-Wallis investigation showed that the number of bacterial colonies in the touch-screen and untouched phones was very different, and the number of bacteria in mechanical type phones was more than touch screen phones (*P* <0.001).

**Table 3:** Average of bacterial colonies in touch and untouched-screen phones (CFU 48 h).

**Descriptive parameters**	**Touch pad**	**Key pad**
Samples	224	232
Average	21.66	35.67
Median	4	22
SD	42.13	81.30
Min	0	0
Max	300	405

The average number of colonies in male students is reported in Table 4. The average number of bacterial colonies in men and women was 35.79 and 21.53 CFU, respectively. The results of the Mann-Whitney study showed that the number of bacteria in the different genders of the subjects was significantly different and the number of bacterial colonies in the cellphones used by men was higher than women (*P* < 0.001).

**Table 4:** Average of colonies in male and female cellphones samples (CFU 48 h).

**Descriptive parameters**	**Male**	**Female**
Samples	214	242
Average	35.79	21.53
Median	14	4
SD	60.04	35.80
Min	0	0
Max	405	280

## 2 Study design, materials and methods

This study was cross-sectional study and performed at the Gonabad University of Medical Sciences (GUMS) in 2016. Totally, 456 cell phone samples from hospital and non-hospital GUMS Staff (232 keypad mobile phones, 224 touch screen phones) were collected according to Cochran׳s sample size formula. A sterile swab moistened with sterile saline was rotated over the surface of both sides of mobile phones. All swabs were immediately streaked onto two plates were contained blood agar supplemented with 5% defibrinated Sheep Blood and eosin methylene blue agar. Plates were incubated aerobically at 37 °C for 48 h. Isolated microorganisms were identified using Gram׳s staining, colony morphology and appropriate biochemical procedures [9], [10], [11], [12]. Finally, data were analyzed using SPSS version 20, the significant *P* < 0.05 was considered. Regarding the un-normal distribution of data (*P* > 0.05), nonparametric tests such as Kruskal Wallis were used to investigation of the differences between various groups. Also, correlation between the logarithm of colony numbers and type of phone analyzed by linear regression analysis.
